# Comparison of a loop-mediated isothermal amplification for orf virus with quantitative real-time PCR

**DOI:** 10.1186/1743-422X-10-138

**Published:** 2013-05-01

**Authors:** Guangxiang Wang, Youjun Shang, Yanhua Wang, Hong Tian, Xiangtao Liu

**Affiliations:** 1 State Key Laboratory of Veterinary Etiological Biology, Lanzhou 730046, China; 2 National Foot and Mouth Disease Reference Laboratory, Lanzhou 730046, China; 3 Key Laboratory of Animal Virology of Ministry of Agriculture, Lanzhou 730046, China; 4 Lanzhou Veterinary Research Institute, Chinese Academy of Agriculture Science, Lanzhou 730046, China

**Keywords:** Orf virus, Loop-mediated isothermal amplification (LAMP), Real-time PCR

## Abstract

**Background:**

Orf virus (ORFV) causes orf (also known as contagious ecthyma or contagious papular dermatitis), a severe infectious skin disease in goats, sheep and other ruminants. Therefore, a rapid, highly specific and accurate method for the diagnosis of ORFV infections is essential to ensure that the appropriate treatments are administered and to reduce economic losses.

**Methods:**

A loop-mediated isothermal amplification (LAMP) assay based on the identification of the F1L gene was developed for the specific detection of ORFV infections. The sensitivity and specificity of the LAMP assay were evaluated, and the effectiveness of this method was compared with that of real-time PCR.

**Results:**

The sensitivity of this assay was determined to be 10 copies of a standard plasmid. Furthermore, no cross-reactivity was found with either capripox virus or FMDV. The LAMP and real-time PCR assays were both able to detect intracutaneous- and cohabitation-infection samples, with a concordance of 97.83%. LAMP demonstrated a sensitivity of 89.13%.

**Conclusion:**

The LAMP assay is a highly efficient and practical method for detecting ORFV infection. This LAMP method shows great potential for monitoring the prevalence of orf, and it could prove to be a powerful supplemental tool for current diagnostic methods.

## Background

The orf virus (ORFV) is the prototype member of the Parapoxvirus genus within the Poxviridae family. The ORFV has a worldwide distribution and causes an infectious skin disease known as contagious ecthyma in goats, sheep and other ruminants [1]. For susceptible young sheep in an epidemic situation, mortality can reach 90% [2]. Therefore, a practical and reliable method for the diagnosis of ORFV infections is required.

For diagnosis of such infections, clinical signs, virus isolation and electron microscopy are commonly used along with serological tests. However, these methods are laborious, time-consuming and, in some cases, not effective. For example, virus isolation can be unsuccessful at times, even when virus-like particles are observed in the lesions resulting from infection. Goats and sheep are commonly infected with the virus, yet serological tests have not confirmed the cause of clinical signs. PCR-based diagnostic assays have been developed for the sensitive and specific detection of ORFV infections [3-7]. However, these assays have not been widely adopted in resource-poor regions due to their relatively complex nature, as well as the need for both expensive equipment and highly trained personnel [8,9].

Loop-mediated isothermal amplification (LAMP) is a simple technique that rapidly amplifies specific DNA sequences with high sensitivity under isothermal conditions [10]. LAMP products can easily be detected by the naked eye due to the formation of magnesium pyrophosphate, a turbid white by-product of DNA amplification that accumulates as the reaction progresses [11]. In addition, LAMP products can be detected by direct fluorescence [12]. Other fluorescent dyes, such as ethidium bromide, SYBR green and Evagreen, have also been used for visualization of LAMP products [13]. Furthermore, Thekisoe et al. have reported that LAMP reagents are relatively stable even when stored at 25 or 37°C, which supports the use of LAMP in field conditions and resource-poor laboratories [14]. Recently, LAMP assays targeting the B2L and DNA polymerase genes of ORFV have been developed, and these methods were found to be powerful diagnostic tools [8,9].

The F1L gene is part of the highly conserved central region of the ORFV genome [15,16]. In the present study, a LAMP assay was developed to specifically identify the F1L gene sequence to facilitate the detection of ORFV infections and the effectiveness of this method was compared with that of real-time PCR.

## Results

### Detection of LAMP product

**Figure 1:**
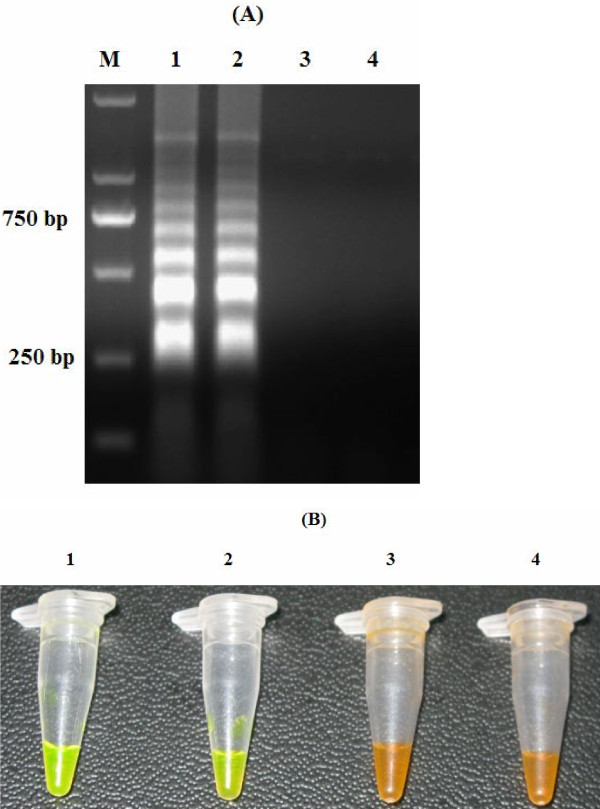
**Analysis of LAMP products.** LAMP amplification products were analyzed using agarose gel electrophoresis **(A)** and visually inspected with SYBR green I dye under daylight conditions against a black background **(B).** M: DNA marker; 1: Plasmid containing the F1L gene; 2: Genomic DNA of ORFV; 3: DNA from healthy goats; 4: Water.

The LAMP products were electrophoresed on a 1.5% agarose gel stained with ethidium bromide solution and visualized under UV light. In addition, visual inspection of the LAMP products was performed by adding SYBR Green I to the reaction mixture tube and observing the fluorescent signals of the solutions under daylight conditions against a black background (Figure 1).

### Sensitivity of LAMP

**Figure 2:**
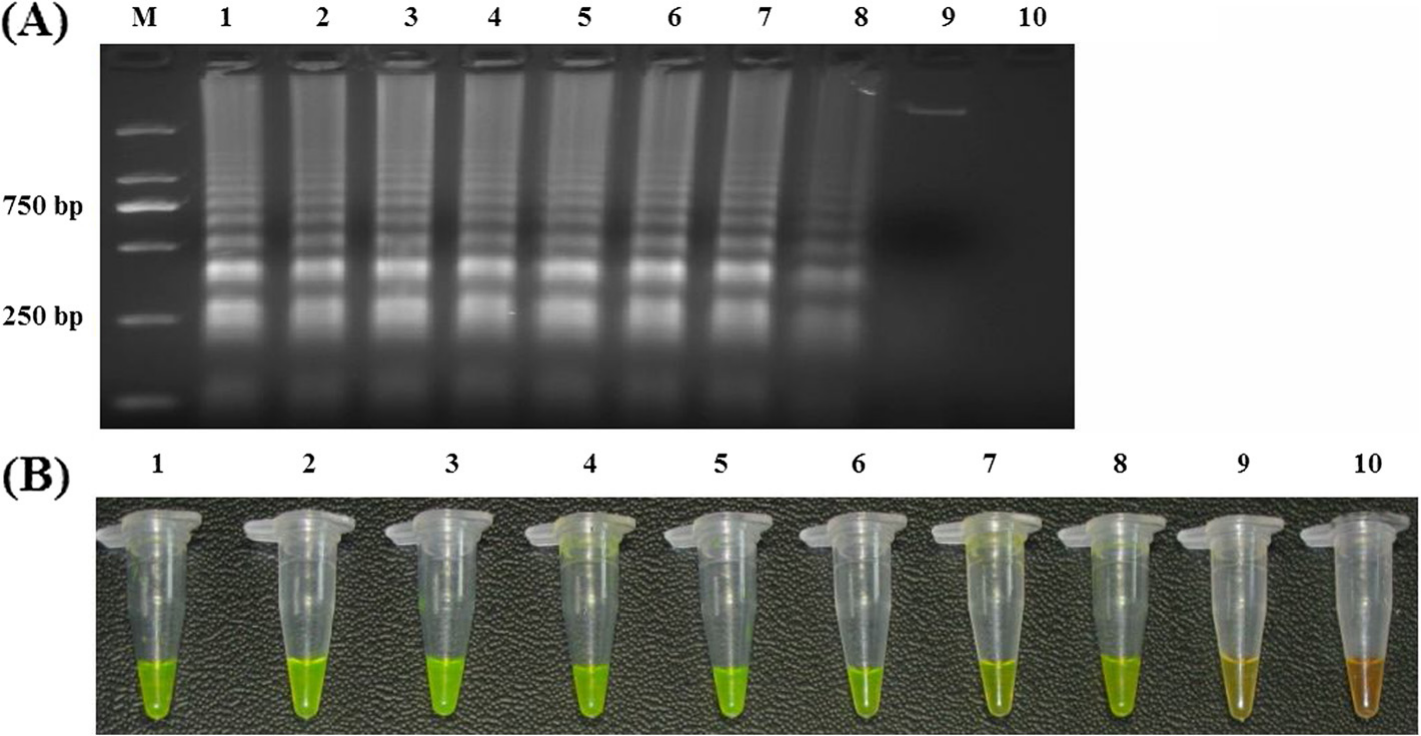
**Sensitivity of LAMP.** Agarose gel electrophoresis **(A)** and visual inspection using SYBR Green I staining of the LAMP products **(B)**. M: DNA marker; 1–9 are the reaction results from a 10-fold serial dilution of plasmid containing the F1L gene from 10^8^ to 10^0^ copies per reaction; 10: Negative control.

To determine the detection limit of the LAMP assay, 10-fold serial dilutions of the PF1L were amplified using LAMP. As determined using both 1.5% agarose gel electrophoresis and color inspection with SYBR Green I dye, the detection limit of the LAMP assay was determined to be 10 copies of DNA (Figure 2).

### Specificity of LAMP

**Figure 3:**
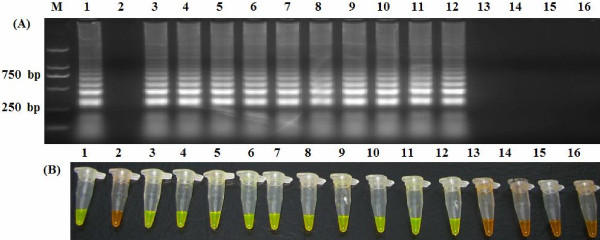
**Specificity of LAMP.** Agarose gel electrophoresis **(A)** and visual inspection using SYBR Green I staining **(B).** M: DNA marker; 1: Plasmid containing the F1L gene; 2: Negative control; 3–12: ORFV/HB/CHA, ORFV/Vaccine/CHA, ORFV/Xinjiang/CHA, ORFV/Chongqing/CHA, ORFV/Shanxi/CHA, ORFV/Guangxi/CHA, ORFV/Gansu/CHA, ORFV/Liaoning/CHA, ORFV/Jilin/CHA and ORFV/Sichuan/CHA; 13 and 14: FMDV/O/CHA and FMDV/Asia I/JS; 15 and 16: Capripox virus/China Vaccine and Capripox virus/Henan/CHA.

The specificity of the LAMP assay was evaluated using the genomic DNA of 10 known ORFV isolates, capripox virus and FMDV. Only the specific ORFV target DNA was amplified by LAMP. No cross-reactivity was observed with the DNA samples of capripox virus or FMDV. These results confirm that the LAMP assay is highly specific (Figure 3).

### Sensitivity and specificity of real-time PCR

**Figure 4:**
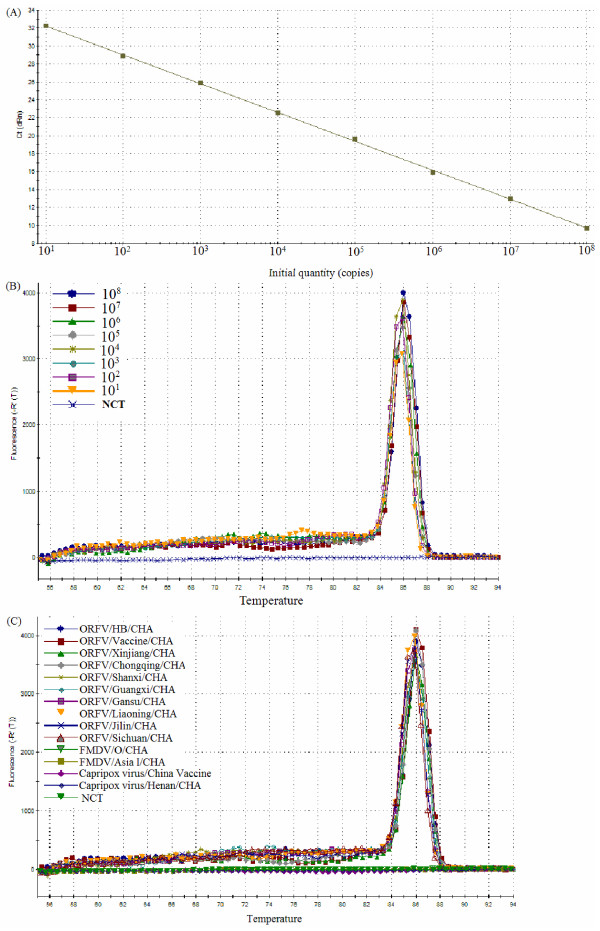
**Sensitivity and specificity of real-time PCR assay.** The standard curve **(A)** and dissociation curve **(B)** were generated using known concentration of recombinant plasmid containing the B2L gene from 10^8^ to 10^0^ copies. The dissociation curves of real-time PCR used to detect ORFV and other select viruses **(C)**.

To determine the detection limit of the real-time PCR, the copy number of a recombinant plasmid containing the B2L gene (PB2L), which was previously constructed by our lab, was calculated as described below. PB2L was amplified from 10-fold serial dilutions using real-time PCR. The detection limit was found to be 10 copies/μl. The real-time PCR slope was −3.218, with an R^2^ = 1 and a reaction efficiency of 104.5%. Moreover, the standard curve generated using the 10-fold serial dilutions of the plasmid was linear over eight orders of magnitude (1 to 10^8^ copies/μl), demonstrating that real-time PCR can be used to accurately quantify this target DNA over a large range of concentrations (Figure 4A).

The primers chosen for real-time PCR were initially validated by monitoring product amplification with SYBR Green I. Melting curve analysis showed a unique peak at 86°C, indicating the formation of a single PCR product without the presence of nonspecific amplification products or primer dimers (Figure 4B). To further determine the specificity of the amplification reaction with the chosen primers, the SYBR Green I-based real-time PCR was performed using DNA from 10 different isolates of ORFV, capripox virus and FMDV. All ORFV DNA samples tested using SYBR Green I-based real-time PCR yielded a positive result. However, the DNA samples from capripox virus and FMDV did not yield an amplification signals (Figure 4C).

### Evaluation of the LAMP assay using samples from experimentally infected goats

To evaluate the practicality and efficiency of the LAMP assay, its ability to detect ORFV infections was tested using the skin lesions from experimentally infected goats. Forty-six samples of skin lesions from four experimentally infected goats were tested using the LAMP (by both visual inspection and agarose gel electrophoresis methods) and real-time PCR assays. Forty-one samples were determined to be positive by both LAMP and real-time PCR. One sample was determined to be negative by LAMP assay but positive by real-time PCR. The sensitivities of the LAMP and real-time PCR assays were 89.13% and 91.30%, respectively (Table 1), and the results were very similar between the two assays.

**Table 1:** Comparison of LAMP and real-time PCR for detection of orf from intracutaneous and cohabitation infections

**The route of infection**	**Samples**	**Days after infection**	**Assay**	
			**LAMP**	**real-time PCR**
Intracutaneous	Goat 1	10	+	+
		11	+	+
		12	+	+
		13	+	+
		14	+	+
		15	+	+
		16	+	+
		17	+	+
		18	+	+
		20	+	+
		22	+	+
		25	-	-
		28	+	+
	Goat 2	12	+	+
		13	+	+
		14	+	+
		15	+	+
		16	+	+
		17	+	+
		18	+	+
		19	+	+
		20	+	+
		23	+	+
		25	+	+
		27	+	+
		30	-	+
Cohabitation	Goat 3	18	+	+
		21	+	+
		22	+	+
		23	+	+
		24	+	+
		25	+	+
		26	+	+
		27	-	-
		29	+	+
	Goat 4	17	+	+
		19	+	+
		20	+	+
		21	+	+
		22	+	+
		23	+	+
		24	+	+
		25	+	+
		27	+	+
		29	-	-
		32	-	-

+, Positive. -, Negative.

## Discussion

Orf is distributed throughout many countries [2,17-23], and ORFV infections can cause weight loss and poor development as the disease prevents host animals from feeding. Therefore, the development of a rapid, simple and sensitive detection method for ORFV infections is required. For the diagnosis of ORFV infections, the LAMP assay has many advantages, such as simplicity, rapidity and inexpensiveness, compared with other nucleic acid-based tests [8,9]. In addition, previous reports have demonstrated that the sensitivity of the LAMP is higher than that of conventional PCR or nested PCR for detecting ORFV infections [8,9]. Therefore, this method shows great potential for use in resource-limited veterinary laboratories in developing countries (e.g., China), where many endemic diseases exist. In the present study, we evaluated the sensitivity and specificity of the rapid diagnostic LAMP method.

The simplest way of detecting LAMP products is to visually inspect the white turbidity that results from magnesium pyrophosphate accumulation as a by-product of the reaction [11]. However, a small amount of this white precipitate is not always distinguishable from other white precipitates, such as proteins or carbohydrates that can be derived from the templates. Therefore, existing “field-friendly” LAMP-based detection systems are still imperfect. Previous report has demonstrated that amplified DNA can be stained (using PicoGreen or ethidium bromide) and visualized in solution with the same sensitivity as agarose gel electrophoresis [8]. In this study, we used the dye SYBR Green I to detect the amplified DNA products. Positive and negative reactions could be differentiated by distinctly different colors when viewed under daylight conditions against a black background (Figure 1). Consistent with previous report, this color inspection method was found to have the same detection limit as agarose gel electrophoresis (Figure 2), which should facilitate the rapid screening of samples without the need for gel electrophoresis. These results indicate that visual color inspection of LAMP products using the SYBR Green I dye should be practical under field conditions.

The reliability of a LAMP assay depends largely on the specificity of the primer sets being used. These primer sets in this study—two outer primers (F3 and B3) and two inner primers (FIP and BIP)—allow for the recognition of six sites in the target sequence that are specific to the F1L gene and that are necessary for the LAMP reaction to occur. Based on the sequences of the ORFV F1L gene available in GenBank and the sequences of ORFV isolates from China, we designed and tested several sets of primers using comparative experiments, and the primer sets that yielded the highest specificity and sensitivity in the LAMP assay is reported here. Our LAMP assay showed high specificity and sensitivity, as it yielded positive results for all 10 of the known ORFV isolates but did not amplify the negative control samples (Figure 3).

Samples from experimentally infected goats were tested using both the LAMP and real-time PCR assays. The LAMP assay correctly identified 41 out of 46 samples as positive (89.13%), and the real-time PCR assay correctly identified 42 out of 46 samples as positive (91.30%). ORFV DNA could be detected in most of the intracutaneous- and cohabitation-infection samples from the time of lesion presentation to recovery (Table 1). Therefore, we observed a very good agreement between the LAMP and real-time PCR results. Only one sample was identified as negative by LAMP (both by visual inspection and agarose gel electrophoresis) but as positive by real-time PCR. This discrepancy was most likely due to very low ORFV levels in this sample, which may have been beyond the detection limit of LAMP. This result suggests that the sensitivity of LAMP is slightly lower than that of real-time PCR. However, it is important to consider that real-time PCR is a time-consuming procedure that requires expensive and relatively complicated equipment, including a thermal cycler with real-time monitoring and data-analysis systems. Therefore, this LAMP-based assay has clear advantages over real-time PCR in terms of practicality, and it can be easily used in any standard diagnostic laboratory, particularly in developing countries where the disease is prevalent.

## Conclusions

We show that a LAMP assay based on amplification of the ORFV F1L gene is rapid, highly specific and accurate for the detection of ORFV infections. The sensitivity of this LAMP (89.13%) was higher than previously reported (70% and 74.3%, respectively). Furthermore, this color inspection method with SYBR Green I dye had the same sensitivity as agarose gel electrophoresis, which should facilitate the rapid large-scale screening of samples and the rapid diagnosis of ORFV infections under field conditions without the use of agarose gel electrophoresis. Therefore, we conclude that this LAMP is a practical and reliable method for detecting ORFV infections, and we suggest that this test can be adopted as a powerful supplemental tool to current diagnostic assays.

## Materials and methods

### Intracutaneous and cohabitation infection of goats

Four healthy 12- to 14-week-old goats were selected for this study. The ORFV strain ORFV/HB/09 [22] was propagated in bovine testicular cells using Eagle’s minimal essential medium (Shanghai Gaochuang Medical Science And Technology Co., Ltd, Shanghai, China) containing 10% fetal calf serum (TCID_50_ = 10^-5.3^/0.1 ml), which was used to infect the goats to prepare the ORFV-positive samples. Two goats were inoculated intradermally on oral mucosa with viral supernatant (0.2 ml per goat) and housed individually. After 7 days, each non-inoculated goat was housed with one inoculated goat, thereby making two replicate cohabitation groups. The clinical signs and macroscopic lesions of goats infected via both routes were observed. The samples of the skin lesions around the muzzle and lips were collected from the time of lesion presentation to recovery, and these samples were used to evaluate of the LAMP method. All experimental procedures and animal care were conducted in accordance with the guidelines and the regulations of the Gansu Animal Care and Use Committee. The experimental protocol was approved by the Ethical Committee of the Lanzhou Veterinary Research Institute, Chinese Academy of Agricultural Sciences (XYXK- (Gan) 2010–003).

### DNA extraction

Briefly, the tissue sample (25 mg) was mechanically homogenized in 250 μl of phosphate-buffered saline (PBS) in a tube using a pellet pestle device. The homogenates were centrifuged at 2000 ×*g* for 3 min, and the supernatant was collected. The DNA templates for the LAMP and RT-PCR assays were extracted using the Universal Genomic DNA Extraction kit (TaKaRa Biotechnology Co., Ltd, Dalian, China) according to the manufacturer’s protocol. DNA samples extracted from healthy goats were used in parallel with the experimental samples as negative controls.

### LAMP assay

The LAMP primer sets (Table 2) were designed from the F1L gene region of the published sequence of ORFV isolate Jilin-Nongan (GenBank accession no. JQ271535.1) using the Primer Explorer V4 Software (http://primerexplorer.jp/elamp4.0.0/index.html). The LAMP reaction was carried out as previously described by Thekisoe et al. [24], with minor modifications. The LAMP reaction mixture with a total volume of 25 μl contained: 12.5 μl of 2 × LAMP reaction buffer (40 mM Tris–HCl [pH 8.8], 20 mM KCl, 16 mM MgSO_4_, 20 mM (NH_4_)_2_SO_4_, 0.2% Tween 20, 1.6 M Betaine, 2.8 mM of each dNTP), 1 μl (8 units) of Bst DNA polymerase (New England Biolabs, Massachusetts, USA), 2.6 μl primer mix (forward inner primer (FIP) and backward inner primer (BIP) at 40 pmol each, forward outer primer (F3) and forward outer primer (B3) at 10 pmol each), 3 μl of template DNA and 5.9 μl of double distilled water. LAMP was performed at 63°C in a water bath for 1 hour, and samples were identified as positive by agarose gel electrophoresis. LAMP reactions were also stained with SYBR green I dye (TaKaRa Biotechnology Co., Ltd, Dalian, China), and samples were identified as positive by a specific color change when viewed under daylight conditions against a black background.

**Table 2:** Sequence of primers designed for LAMP amplification of the F1L gene of ORFV

**Primer name**	**Sequence (5′-3′)**	**Amplicon size (F2–B2c)**
F3	GGTGGTCTACCCCGAGTAC	782–961^a^
B3	GGCGATGAACCACAGCAG	
FIP (F1c + F2)	TAGATGGCGCCGGGCCAGA-AAACGCGGCTCAGCGG	
BIP (B1c + B2)	CTCCTTCATGGGGCTCTTCGAC-GCAGCAGCAGCACGAG	

^a^Nucleotide position based on the sequence of F1L gene region of ORFV isolate Jilin-Nongan (GenBank accession no. JQ271535.1).

### Sensitivity and specificity of the LAMP assay

The copy number of the recombinant plasmid containing the F1L gene (PF1L), which was constructed previously by our lab, was calculated as described by Wang et al. [25]. Briefly, the concentration of PF1L (X) was determined by spectrophotometry and converted to number of molecules using the following formula: copies/ml = 6.02 × 10^23^ × X/Y (X = OD_260_ × 50 × 10^-6^ g/ml × dilution factor; Y = the length of PF1L (bp) × 660). The sensitivity of the LAMP assay was then determined using the 10-fold serial dilutions of PF1L. Additionally, the specificity of the LAMP assay was determined using DNA from 10 known isolates of ORFV, 2 strains of capripox virus and 2 strains of FMDV.

### Real-time PCR

The primers for SYBR Green I-based real-time PCR were synthesized according to the published reference [7], 5′-CAGCAGAGCCGCGTGAA-3′, and 5′-CATGAACCGCTACAACACCTTCT-3′. Real-time PCR was performed with the detection of the B2L gene of ORFV on an ABI PRISM 7500 thermocycler. The real-time PCR reactions were prepared for a 25 μl reaction volume containing 2 × SYBR Premix Ex Taq II supplemented with ROX II, the primers (10 μM each) and 3 μl of DNA template. The cycling parameters were as follows: preheat at 95°C for 30 s, then 40 cycles of 95°C for 5 s and 64°C for 20 s. After amplification, the data were then analyzed using the 7500 System software. A melting curve analysis was performed to verify the uniqueness of the amplified product by its specific melting temperature.

## Competing interests

The authors declare that they have no competing interests.

## Authors’ contributions

GXW, YJS and XTL designed the experiment. GXW performed lab work. GXW and YHW participated in data analysis and drafted the manuscript. XTL, YJS and HT revised the manuscript. All the authors read and approved the final manuscript.
